# Post-thyroidectomy hypocalcemia exacerbated by chyle leak

**DOI:** 10.1530/EDM-14-0110

**Published:** 2015-03-01

**Authors:** Naweed Alzaman, Anastassios G Pittas, Miriam O'Leary, Lisa Ceglia

**Affiliations:** 1 Division of Endocrinology, Diabetes, and Metabolism, Tufts Medical Center, 800 Washington Street, Boston, Massachusetts, 02111, USA; 2 Department of Otolaryngology, Tufts Medical Center, 800 Washington Street, Boston, Massachusetts, 02111, USA

## Abstract

Transient hypocalcemia after thyroidectomy is not uncommon and the risk increases with the extent of neck surgery. We report a case of severe and prolonged hypocalcemia after total thyroidectomy complicated by thoracic duct injury. Hypoparathyroidism and thoracic duct injury are potential complications following total thyroidectomy with extensive lymph node dissection. This case suggested that having both conditions may complicate treatment of hypoparathyroid-induced hypocalcemia by way of losses of calcium and vitamin D in the chyle leak.

**Learning points:**

- This report highlights chyle leak as an uncommon cause of prolonged hypocalcemia in patients who have undergone extensive neck surgery.
- Chyle has an electrolyte concentration similar to that of plasma.
- Medical treatment options for a chyle leak include fat-free oral diet or parenteral nutrition without oral intake, pharmacological treatment (primarily octreotide).

## Background

Patients who undergo total thyroidectomy and/or extensive neck dissection are at a higher risk for hypocalcemia. One common cause is acute hypoparathyroidism because of devascularization and/or inadvertent removal of parathyroid glands during neck dissection (1). Although not a well-known cause of hypocalcemia, injury to the thoracic duct causing a leak of chyle is a common complication of extensive neck surgery (2) (3). Chyle leakage develops as a result of injury to the lymphatic duct entering the venous system at the junction of the internal jugular and subclavian veins during or after lateral neck dissection (8). Its injury has been associated with loss of electrolytes and fat-soluble vitamins.

We report a case of a patient who underwent total thyroidectomy with extensive neck dissection for medullary thyroid cancer. Surgery was complicated by hypoparathyroidism and thoracic duct injury. Post-operatively, hypocalcemia was treated with oral calcium and calcitriol; however, it abruptly worsened requiring continuous i.v. calcium infusion during a concurrent increase in the output of chyle from the injured thoracic duct. Stabilization of serum calcium concentration with transition to oral calcium and calcitriol occurred simultaneously with a decrease in the chyle output.

## Case presentation

A 58-year-old man with multiple thyroid nodules and enlarged cervical lymph nodes was diagnosed with medullary thyroid cancer via fine needle aspiration biopsy. The patient underwent total thyroidectomy with extensive lymph node dissection that was complicated by hypoparathyroidism and thoracic duct injury.

## Investigation

**Figure 1:**
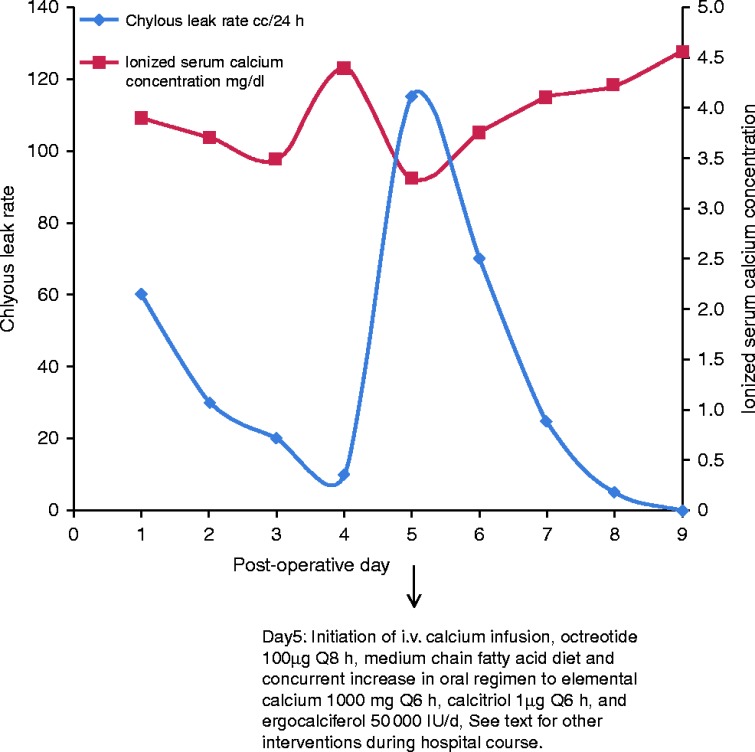
Association between ionized calcium concentration and the rate of chyle leak.

On post-operative day 1, serum ionized calcium (iCa) level was 3.9 mg/dl (4.2-5.2 reference range) and parathyroid hormone (PTH) was <3 pg/ml. After receiving 2 ampoules of intravenous (i.v.) calcium gluconate (186 mg of elemental calcium), the patient was started on 3 g oral calcium carbonate (CaCO_3_), calcitriol 0.5 μg, and vitamin D_3_ 1000 IU daily. In the first three post-operative days, oral calcium and vitamin D treatment was increased and an additional bolus of calcium gluconate was required. However, on post-operative day 5, he developed symptoms of tetany (peri-oral numbness and a Chvostek's sign), confusion, and dysarthria in spite of the oral medication regimen. Serum iCa had declined from 4.38 to 3.29 mg/dl and serum 25-hydroxyvitamin D level was found to be low at 17 ng/ml. He was transferred to the intensive care unit and placed on a continuous intravenous calcium gluconate infusion. In the evaluation of the refractory hypocalcaemia, it was noted that the significant decline in serum calcium concentration coincided with an increased rate of output of chyle originating from the thoracic duct injury (see Fig. 1). At that time on post-operative day 5, serum Ca was at its lowest level and the rate of the chyle output was at its maximum at 115 cc over 24 hours. Biochemical analysis of the chyle revealed a total calcium concentration of 5.3 mg/dl. Circulating 25-hydroxyvitamin D and its active 1, 25-dihydroxyvitamin D were not measured, but are typically present in chyle.

## Treatment

Over the following five days, doses of calcium carbonate were increased to 4 g, calcitriol to 4 μg, and vitamin D_2_ to 50 000 IU daily. The chyle leak was managed by initiating both a medium chain fatty acid diet, which contained a low fat content, and subcutaneous octreotide 100 microgram three times daily for 10 days. 

## Outcome and follow-up

Subsequently the chyle output decreased to <15 cc/day and serum iCa level rose to 4.6 mg/dl. Calcium levels remained stable off of i.v. calcium and only on oral supplementation. Serum iCa level gradually improved to a range from 3.7 to 4.2 mg/dl over the ensuing 3 days, and tetany symptoms resolved.

## Discussion

The main cause of hypocalcemia in this patient was post-surgical hypoparathyroidism; however, our patient's abrupt worsening of hypocalcemia may have been exacerbated by the calcium and vitamin D losses from a leak of chyle from an injured thoracic duct. Notably, as the rate of chyle output declined with nutrition modification and medical therapy, serum iCa improved and supplementation requirements declined.

Chyle is an odorless, alkaline fluid. Approximately 2–4 l of chyle are produced each day. Approximately 70% of chyle is absorbed dietary fat, mainly in the form of triglycerides. Chyle has an electrolyte concentration similar to that of plasma (3). Leaks of chyle fluid commonly result in nutritional deficiencies due to the loss of calories (200/l), protein, and fat-soluble vitamins. Metabolic complications including hypocalcemia may also occur due to the loss of fluid and electrolytes (4). Treatment options for a chyle leak include drainage, conservative therapy with nutrition intervention with fat-free oral diet, or parenteral nutrition without oral intake, pharmacological treatment (primarily octreotide), and finally direct surgical repair. Prior reports indicate that use of octreotide, a potent inhibitor of gastrointestinal hormones including gastrin, motilin, secretin, and pancreatic polypeptide, is also safe and effective (5) (6) (7).

In this patient with permanent hypoparathyroidism and thoracic duct injury, the calcium level required frequent close monitoring because of worsening hypocalcemia. A combination of aggressive i.v./oral calcium and vitamin D replacement in addition to initiation of medium-chain fatty acid diet and s.c. octreotide may be needed to manage hypocalcemia in the setting of a chyle leak.

## Patient consent

Written informed consent has been obtained from the patient for publication of this article.

## Author contribution statement

Dr N Alzaman, Dr A G Pittas, and Dr L Ceglia helped to manage hypoparathyroidism and contributed to the writing of this manuscript. Dr M O'Leary performed total thyroidectomy with central neck dissection and contributed towards managing chyle leak. Dr. O'Leary reviewed and approved the manuscript.
